# Defects, dopants and Mg diffusion in MgTiO_3_

**DOI:** 10.1038/s41598-019-40878-y

**Published:** 2019-03-13

**Authors:** Navaratnarajah Kuganathan, Poobalasuntharam Iyngaran, Ruslan Vovk, Alexander Chroneos

**Affiliations:** 1 Department of Materials, Imperial College London, London, SW7 2AZ United Kingdom; 2 Department of Chemistry, University of Jaffna, Sir. Pon Ramanathan Road, Thirunelvely, Jaffna Sri Lanka; 3 Physics Department, V. Karazin Kharkiv National University, Svobody Sq. 4, 61077 Kharkiv, Ukraine; 4 Faculty of Engineering, Environment and Computing, Coventry University, Priory Street, Coventry, CV1 5FB United Kingdom

## Abstract

Magnesium titanate is technologically important due to its excellent dielectric properties required in wireless communication system. Using atomistic simulation based on the classical pair potentials we study the defect chemistry, Mg and O diffusion and a variety of dopant incorporation at Mg and Ti sites. The defect calculations suggest that cation anti-site defect is the most favourable defect process. The Mg Frenkel is the second most favourable intrinsic defect though the formation energy is highly endoergic. Higher overall activation energies (>3 eV) are observed for oxygen migration compared to those observed for magnesium (0.88 eV). Dopant substitution energies for a range of cations with charges varying from +2 to +4 were examined. Divalent dopants (Mn^2+^, Fe^2+^, Co^2+^, Ca^2+^ and Zn^2+^) on the Mg site exhibit low solution energies. Trivalent dopants prefer to occupy Mg site though their solution energies are high. Exothermic solution energies calculated for tetravalent dopants (Ge^4+^ and Si^4+^) on the Ti site suggest the necessity of experimental verification.

## Introduction

Ilmenite titanates such as MgTiO_3_ are technologically important as they have low dielectric loss and high dielectric constant^1–8^. Materials with low dielectric loss are useful as dielectric resonators in integrated circuits for wireless networks, global positioning systems and mobile phones^6^.

Understanding self-diffusion, the intrinsic and extrinsic defect processes can be important for the optimization of the properties of most classes of materials including semiconductors and oxides^9–11^. Defect engineering strategies where isovalent or aliovalent dopants are introduced in the lattice are commonly used in ceramics. The solution of ions, at the Mg site, with different ionic size can improve the dielectric properties and signal-to-noise ratio of MgTiO_3_. Conventional solid experimental method was used by Zhang *et al*.^5^ to synthesis (Mg_0_._97_M_0.03_)TiO_3_ (M = Ni, Zn, Co and Mn) ceramics and its structural and dielectric properties have been discussed. Using the semialkoxide precursor method Kumar *et al*.^6^ reported the synthesis of cobalt doped MgTiO_3_ concluded that (Mg_0_._95_Co_0_._05_)TiO_3_ ceramic is a promising dielectric material. From a physical viewpoint the incorporation of dopants at the Mg site can affect the bonding, polarizability and the octahedral distortion and these in principle can modify the dielectric properties in MgTiO_3_. There are only limited studies on the impact of dopant solution in MgTiO_3_ and the comprehensive theoretical understanding of these defect processes is presently lacking. In essence computational modelling can guide experimental work to the most appropriate doping strategies. Additionally, computational modelling can effectively provide energy trends and valuable insights on the diffusion mechanism and energetics that can complement experiment.

In the present study we have employed atomistic simulations study the intrinsic defect processes, Mg and O diffusion, solution of MO (M = Ni, Cu, Zn, Co, Fe, Mn, Ca, Sr and Ba), M_2_O_3_ (M = Al, Mn, Co, Mn, Sc and Yb) and MO_2_ (M = Si, Ge, Sn, Zr and Ce).

## Results and Discussion

### MgTiO_3_ structure

**Figure 1:**
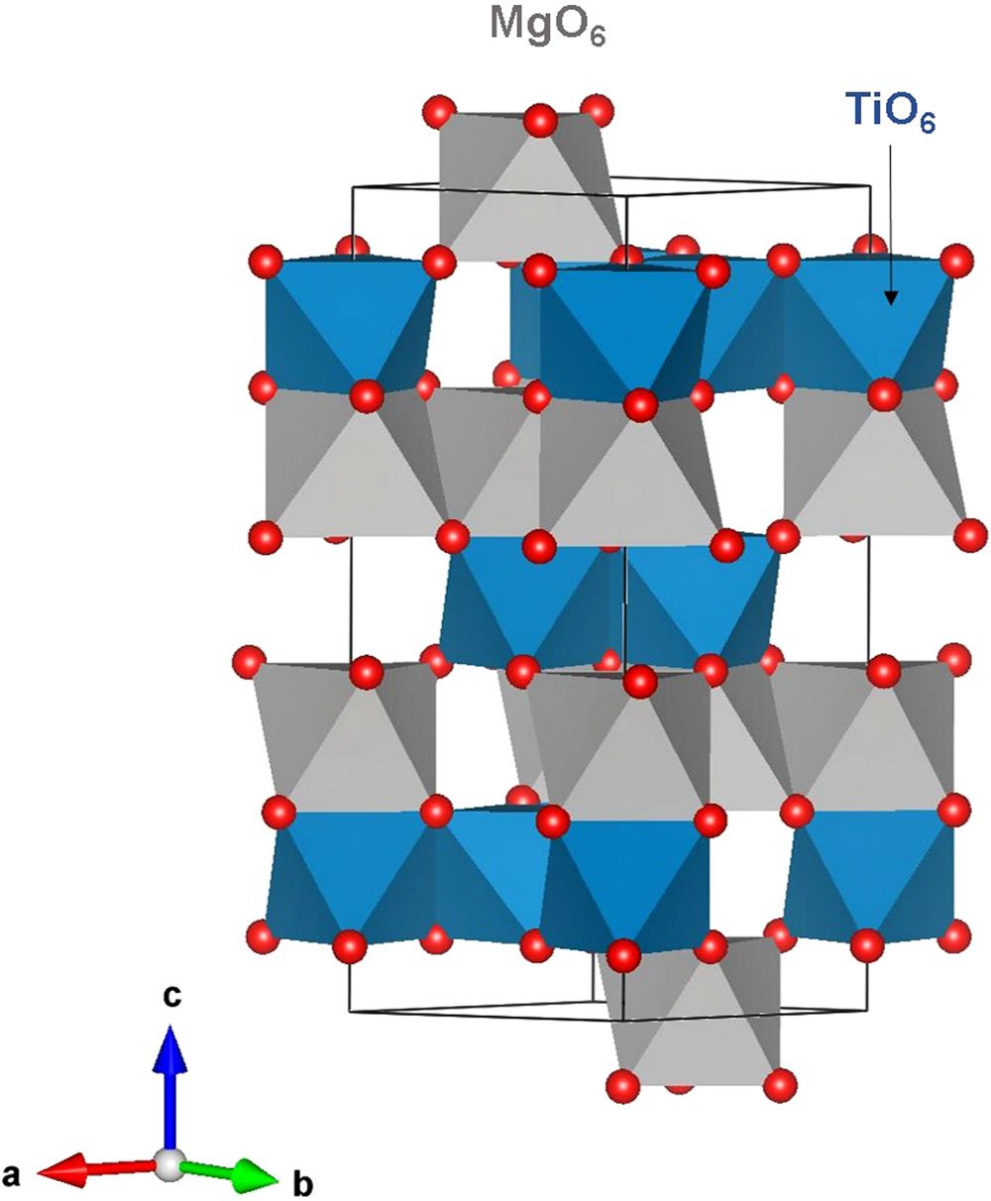
Crystal structure of MgTiO_3_ (space group $R\bar{3}H$).

Figure 1 represents the crystal structure of rhombohedral MgTiO_3_ (space group $R\bar{3}H$, lattice parameters a = b = 5.05478 Å, c = 13.8992 Å, α = 90°, β = 90° and γ = 120°) as observed in the experiment by Wechsler *et al*.^12^. The cations are surrounded by six O^2−^ ions forming MO_6_ octahedrons (M = Mg and Ti) which form layers in the *ab* plane (refer to Fig. 1). Using atomistic simulation based on the classical potentials we reproduced the complex experimental structure with the available pair potentials published in the literature (refer to Table S1 in the Supplementary Potential Information). The calculated structural parameters are in good agreement with the experimental values within a margin of 2% error (refer to Table 1).

**Table 1:** Calculated structural parameters and corresponding experimental values reported for rhombohedral $(R\bar{3}H)$ MgTiO_3_.

Parameter	Calc	Expt^12^	|∆|(%)
a (Å)	5.125035	5.05478	1.39
b (Å)	5.125035	5.05478	1.39
c (Å)	13.626325	13.8992	1.96
α (°)	90.0000	90.0000	0.00
β (°)	90.0000	90.0000	0.00
γ (°)	120.0000	120.0000	0.00
V (Å^3)^	309.958118	307.55653	0.78

### Intrinsic defect processes

We calculated energies of isolated point defects in MgTiO_3_ and then combined them to derive Frenkel and Schottky energies to understand the electrochemical behaviour in this material. The intrinsic defect reactions for Frenkel, Schottky and anti-site defects in the framework of Kröger-Vink notation^13^ are given below (Eqs 1–8).

1 $${\rm{Mg}}\,{\rm{Frenkel}}:{{\rm{Mg}}}_{{\rm{Mg}}}^{{\rm{X}}}\to {V}_{{\rm{Mg}}}^{{\prime\prime} }+{{\rm{Mg}}}_{{\rm{i}}}^{\bullet \bullet }$$

2 $${\rm{O}}\,{\rm{Frenkel}}:{{\rm{O}}}_{{\rm{O}}}^{{\rm{X}}}\to {V}_{{\rm{O}}}^{\bullet \bullet }+{{\rm{O}}}_{{\rm{i}}}^{{\prime\prime} }$$

3 $${\rm{Ti}}\,{\rm{Frenkel}}:{V}_{{\rm{Ti}}}^{{\rm{X}}}\to {V}_{{\rm{Ti}}}^{{\prime\prime} {\prime\prime} }+{{\rm{Ti}}}_{{\rm{i}}}^{\bullet \bullet \bullet \bullet }$$

4 $${\rm{Schottky}}:{{\rm{Mg}}}_{\mathrm{Mg}\,}^{{\rm{X}}}+{{\rm{Ti}}}_{{\rm{Ti}}}^{X\,}+3{{\rm{O}}}_{{\rm{O}}}^{{\rm{X}}}\to {V}_{{\rm{Mg}}}^{{\prime\prime} }+{V}_{{\rm{Ti}}}^{{\prime\prime} {\prime\prime} }+3{V}_{{\rm{O}}}^{\bullet \bullet }+{{\rm{MgTiO}}}_{3}$$

5 $${\rm{MgO}}\,{\rm{Schottky}}:{{\rm{Mg}}}_{{\rm{Mg}}}^{{\rm{X}}}+{{\rm{O}}}_{{\rm{O}}}^{X\,}\to {V}_{{\rm{Mg}}}^{{\prime\prime} }+{V}_{{\rm{O}}}^{\bullet \bullet }+{\rm{MgO}}$$

6 $${{\rm{TiO}}}_{2}\,{\rm{Schottky}}:{{\rm{Ti}}}_{{\rm{Ti}}}^{{\rm{X}}}+2{{\rm{O}}}_{{\rm{O}}}^{X\,}\to {V}_{{\rm{Ti}}}^{{\prime\prime} {\prime\prime} }+2{V}_{{\rm{O}}}^{\bullet \bullet }+{{\rm{TiO}}}_{2}$$

7 $${\rm{Mg}}/{\rm{Ti}}\,\mathrm{antisite}\,({\rm{isolated}}):{{\rm{Mg}}}_{{\rm{Mg}}}^{{\rm{X}}}+{{\rm{Ti}}}_{{\rm{Ti}}}^{X\,}\to {{\rm{Mg}}}_{{\rm{Ti}}}^{{\prime\prime} }+{{\rm{Ti}}}_{{\rm{Mg}}}^{\bullet \bullet }$$

8 $${\rm{Mg}}/{\rm{Ti}}\,\mathrm{antisite}\,({\rm{cluster}}):{{\rm{Mg}}}_{{\rm{Mg}}}^{{\rm{X}}}+{{\rm{Ti}}}_{{\rm{Ti}}}^{{\rm{X}}}\to {\{{{\rm{Mg}}}_{{\rm{Ti}}}^{{\prime\prime} }:{{\rm{Ti}}}_{{\rm{Mg}}}^{\bullet \bullet }\}}^{{\rm{X}}}$$

**Figure 2:**
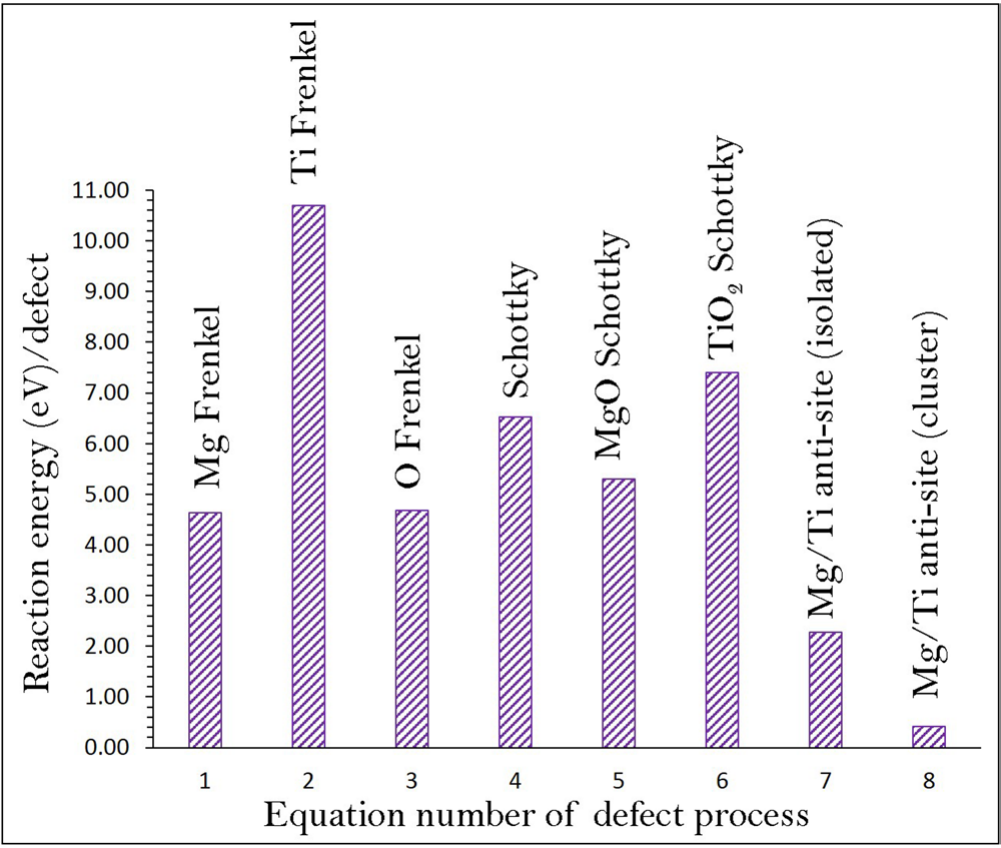
Energetics of intrinsic defect process calculated in rhombohedral MgTiO_3_.

Defect reaction energies for these intrinsic defect processes are reported in Fig. 2 (refer to Table S2). Calculation formulas for these defect process are reported in Table S3. Results suggest that the formation of Mg-Ti anti-site defects (${{\rm{Mg}}}_{{\rm{Ti}}}\text{'}\text{'}\,{\rm{and}}\,{{\rm{Ti}}}_{{\rm{Mg}}}^{\bullet \bullet }$) in the form of cluster (Equation 8) is the lowest energy process (0.42 eV/defect). This indicates a small percentage of cation mixing will be present at high temperatures. This is a typical defect found both experimentally and theoretically in numerous materials^14–28^. The cluster form of anti-site defect (Equation 7) means both defects were created close to each other in the lattice and calculated simultaneously. In the case of isolated form (2.27 eV/defect), defects were calculated independently. There is a reduction in the formation energy of cluster due to the binding of the oppositively charged defects. The second most favourable defect process is the Mg Frenkel (4.63 eV/defect). The O Frenkel is higher only by 0.05 eV/defect than the Mg Frenkel. Nevertheless, these defects are unlikely to occur at low temperatures due to their high endothermic reaction energies. Schottky defect energies are even higher in energy meaning that they are highly unfavourable. The formation enthalpy of MgO via the MgO Schottky-like reaction (relation 5) leading to the formation of further ${V}_{{\rm{Mg}}}^{{\prime\prime} }$ and ${V}_{O}^{\cdot \cdot }$ is calculated to be 5.30 eV per defect, implying that this process can take place at elevated temperatures.

### Self-diffusion

Using the classical pair-potential method it is possible to examine various possible ionic diffusion paths in MgTiO_3_. Diffusion paths are often difficult to observe in experiments and the current methodology has been successfully applied to various ionic materials to establish diffusion paths. The formation of high concentration of vacancies can increase the ionic transport in this material. We therefore examined vacancy assisted migration paths in MgTiO_3_. As Ti Frenkel energy (10.70 eV/defect) is highly endoergic, our calculations considered only Mg and O diffusion.

**Figure 3:**
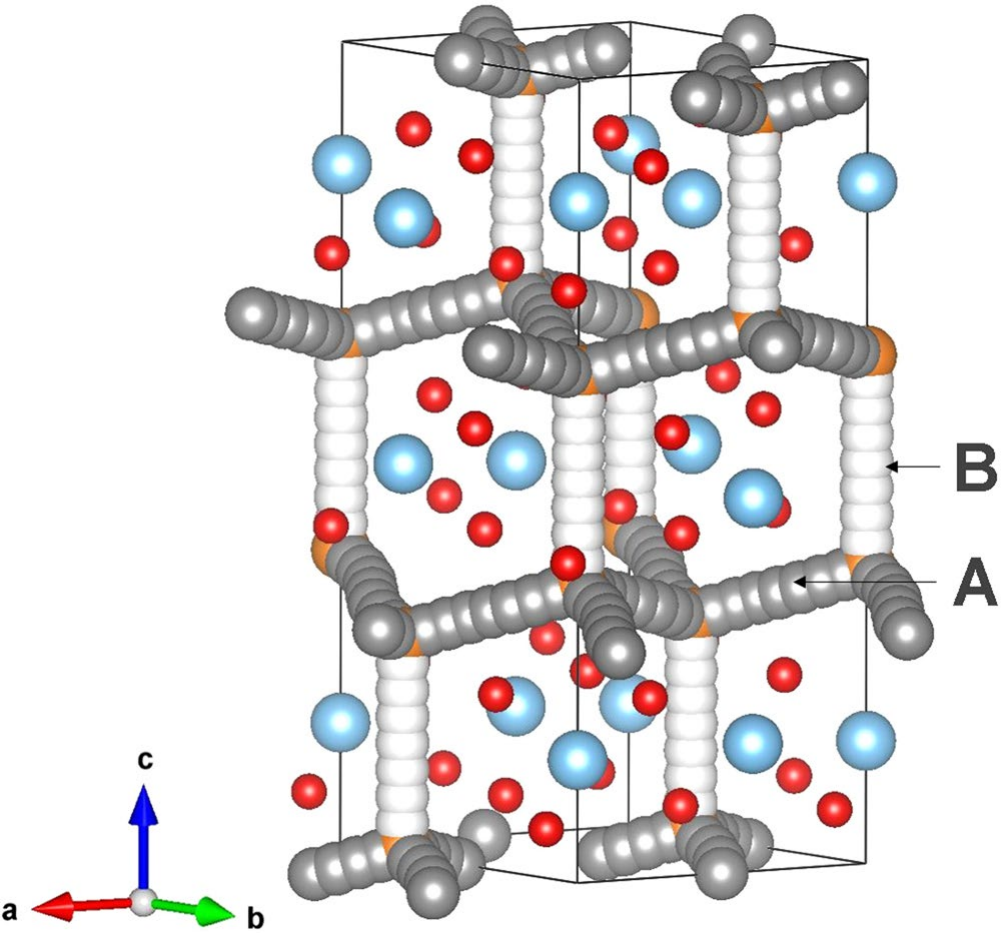
Possible long-range magnesium vacancy migration paths considered. Grey and white colour atoms correspond to different Mg hopping trajectories.

**Figure 4:**
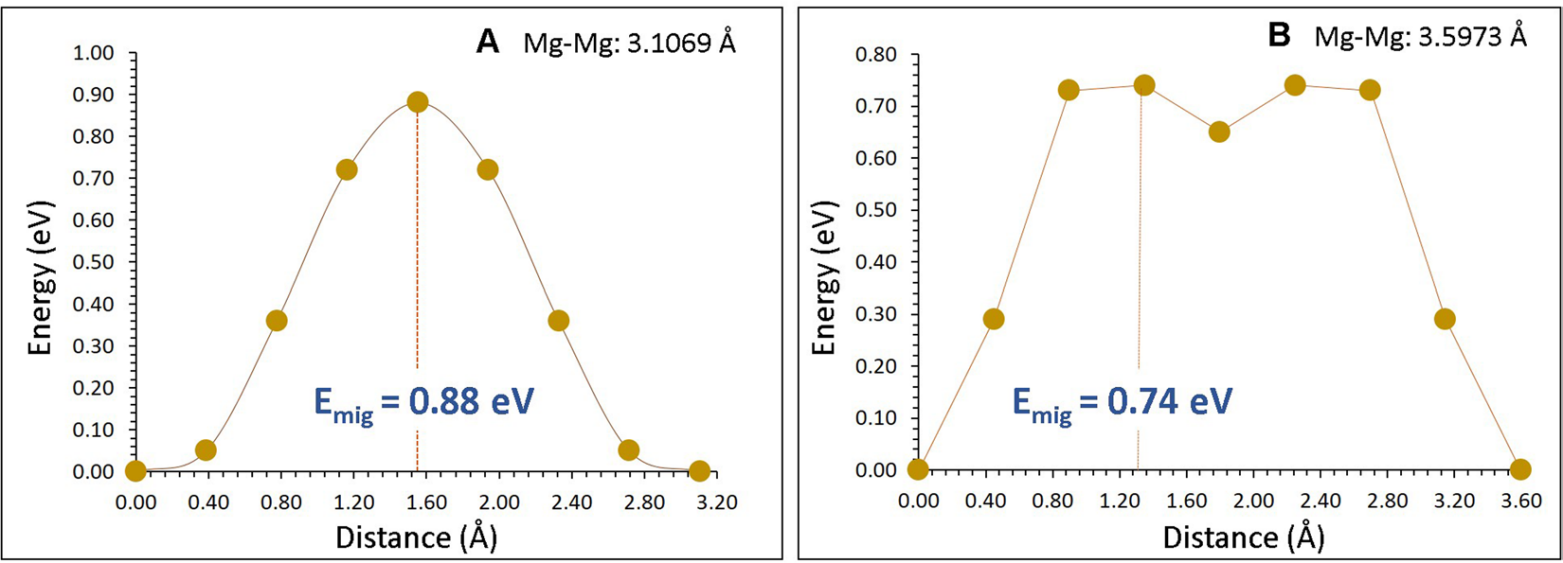
Two different energy profiles [as shown in Fig. 3] of Mg vacancy hopping between two adjacent Mg sites in MgTiO_3_.

For Mg diffusion, two different local Mg hops (refer to Fig. 3) were identified. Migration energies together with the Mg-Mg separation are reported in Table 2. Figure 4 shows energy profile diagrams for each Mg hops. Possible long-range paths connecting local Mg hops with lower overall activation energy were constructed. We identified two long-range three dimensional paths (refer to Fig. 3). The first long range path (along the *ab* plane) exhibits a zig-zag pattern (A→A→A→A) with overall activation energies of 0.88 eV. In the second long-range path (A→B→A→B), the Mg ion migrates along the *c* axis plane with overall migration energy of 0.88 eV, though the activation energy of local Mg hop B is 0.74 eV. There are two identical transition states and an intermediate observed in the local hop B. This is due to the interaction between migrating Mg with equivalent sets of Mg ions present in two different places. These results reveal that Mg ion can migrate via either first or second long range paths.

**Table 2:** Calculated Mg-Mg separations and activation energies using classical pair-potential method for the magnesium ion migration between two adjacent Mg sites (refer to Fig. 3).

Migration path	Mg-Mg separation/Å	Activation energy/eV
A	3.1069	0.88
B	3.5973	0.74

**Figure 5:**
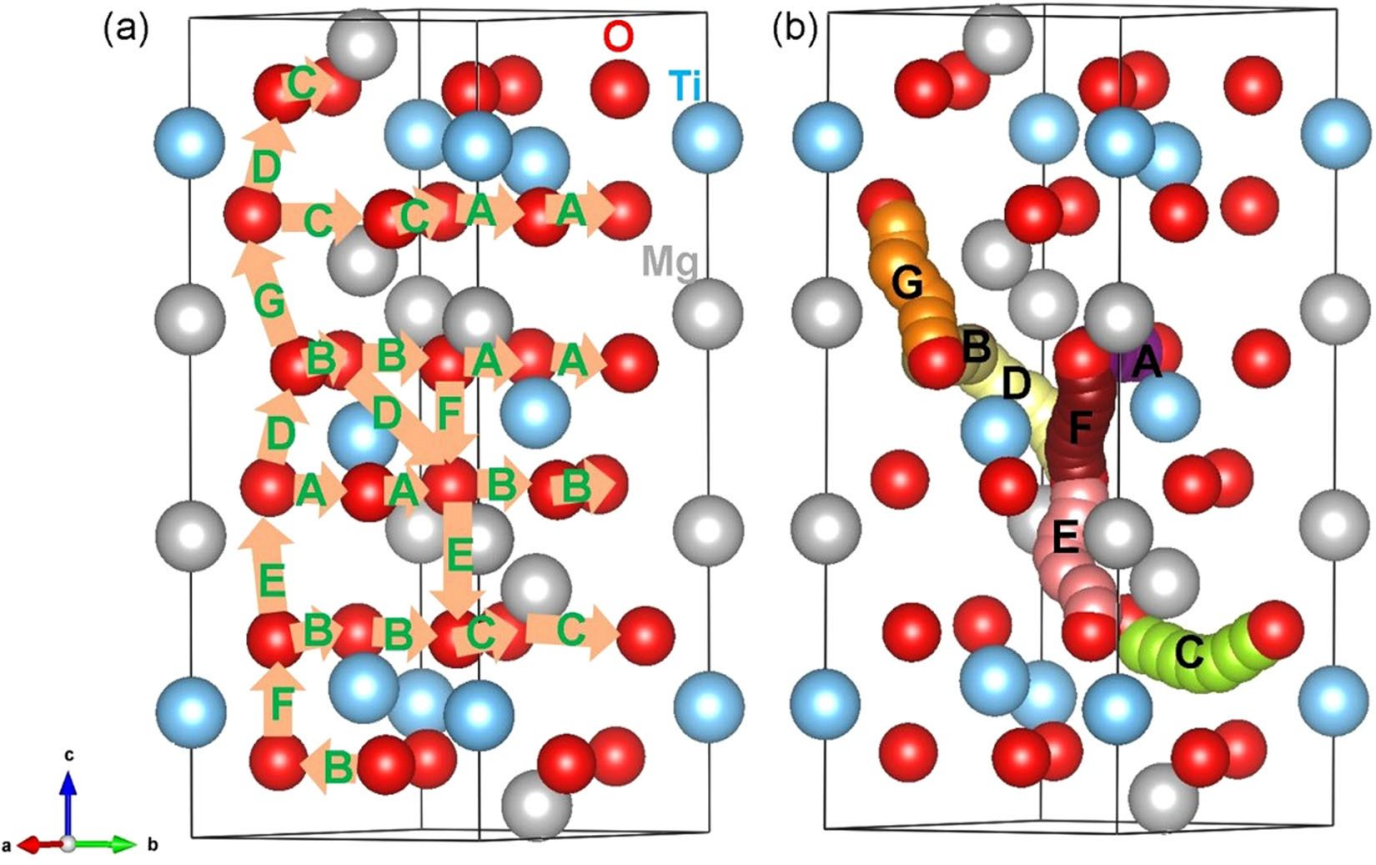
(**a**) Seven possible oxygen vacancy local hops connecting possible long-range migration paths considered and (**b**) local O hopping showing curved trajectories.

**Figure 6:**
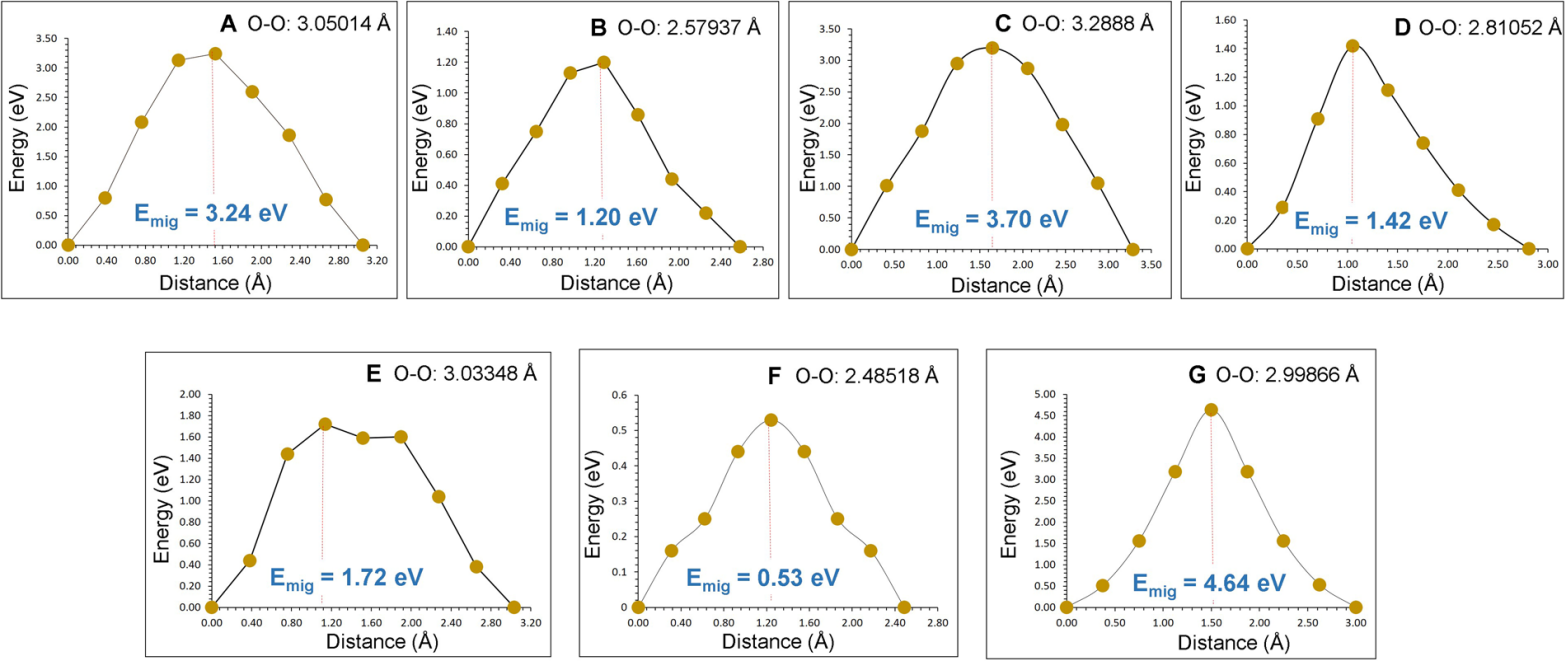
Seven different energy profiles [as shown in Fig. 5] of O vacancy hopping between two adjacent O sites in MgTiO_3_.

Next we calculated various local oxygen migration hops and their corresponding activation energies (refer to Table 3). We identified seven oxygen local hops (A–G) and then constructed three dimensional long range paths as shown in Fig. 5. Energy profile diagrams for local O hops are shown in Fig. 6. In general, higher activation energies are observed for local O hops compared to those calculated for Mg hops. Local O hop F has the lowest activation energy of 0.53 eV. This is due to the shortest O-O separation. Local hops A, B and C are in the *ab* plane with activation energies of 3.24 eV, 1.20 eV and 3.70 eV respectively. In the remaining local hops (D, E, F and G), oxygen atoms are between two adjacent layers. Local hops were then connected to construct long range diffusion paths. Four possible long range paths (refer to Fig. 5) were identified and their overall activation energies are reported in Table 4. Our examination reveals that in all cases the overall activation energy is high (>3 eV) meaning that oxygen ionic conductivity in this material is slow. The long range path (A→A→B→B) with lowest activation energy (3.24 eV) lies in the *ab* plane. The same overall activation energy of 3.70 eV is calculated for long range paths B→B→C→C and C→C→A→A. This is due to the involvement of local hop C which has an activation energy of 3.70 eV. The highest activation energy (4.64 eV) is observed for F→E→D→G→D path in which oxygen migrates along the c axis.

**Table 3:** Calculated O-O separations and activation energies using classical pair-potential method for the oxygen ion migration between two adjacent O sites (refer to Fig. 5).

Migration path	O-O separation/Å	Activation energy/eV
A	3.05014	3.24
B	2.57937	1.20
C	3.28880	3.70
D	2.81052	1.42
E	3.03348	1.72
F	2.48518	0.53
G	2.99866	4.64

**Table 4:** Possible long-range O ion diffusion paths and their corresponding overall activation energies.

Long-range path	Direction	Overall activation energy/eV
A→A→B→B	*ab* plane	3.24
B→B→C→C	*ab* plane	3.70
C→C→A→A	*ab* plane	3.70
F→E→D→G→D	*c* axis	4.64

### Dopant substitution

Here we consider a variety of isovalent and aliovalent dopants on both Mg and Ti sites. In the case of aliovalent substitution, charge-compensation required necessary vacancies and interstitials. In all cases appropriate lattice energies were calculated using the same Buckingham potentials used in this study.

First, divalent dopants were considered on both Mg and Ti sites. The following reaction equations were used to calculate solution energies:

M^2+^ dopants on Mg

9 $${\rm{MO}}+{{\rm{Mg}}}_{\mathrm{Mg}\,}^{{\rm{X}}}\to {{\rm{M}}}_{\mathrm{Mg}\,}^{{\rm{X}}}+{\rm{MgO}}$$

M^2+^ dopants on Ti

10 $${\rm{MO}}+{{\rm{Ti}}}_{\mathrm{Ti}\,}^{{\rm{X}}}+{{\rm{O}}}_{O\,}^{{\rm{X}}}\to {{\rm{M}}}_{\mathrm{Ti}\,}^{{\prime\prime} }+{{\rm{V}}}_{O\,}^{\bullet \bullet }+{{\rm{TiO}}}_{2}$$

**Figure 7:**
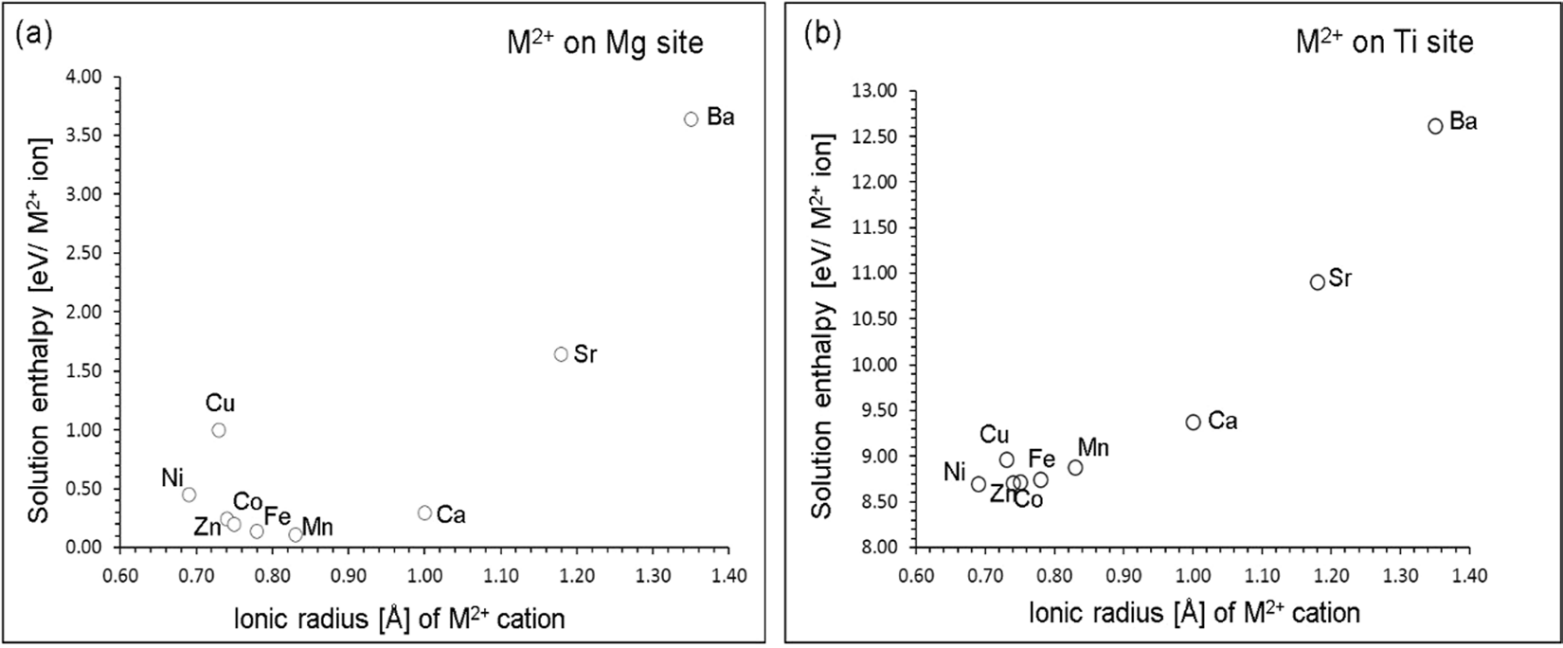
Enthalpy of solution of MO (M = Ni, Cu, Zn, Co, Fe, Mn, Ca, Sr and Ba) with respect to the M^2+^ ionic radius in MgTiO_3_.

Favourable solution energies (<0.30 eV, refer to Table S4) were noted for Mn, Fe, Co, Zn and Ca on Mg site (refer to Fig. 7a). Ni exhibits a slightly higher solution energy (0.45 eV/defect). The current result is in agreement with the successful experimental preparations of (Mg_0_._97_M_0.03_)TiO_3_ (M = Ni, Zn, Co and Mn)^5^ and (Mg_0_._95_Co_0_._05_)TiO_3_^6^ ceramics. Further increase in solution energies is observed for Cu and Sr. The incorporation of Ba^2+^ is highly unfavourable suggesting that this process is unlikely to occur. In the case of M^2+^ ions on Ti site, solution energies are greater than 8 eV (refer to Fig. 7b). Thus, incorporation of M^2+^ ions on Ti site is highly unlikely.

Next, we considered the incorporation of M^3+^ ions on Mg and Ti sites with vacancies and interstitials as charge-compensating defects as defined by the following equations:

M^3+^ dopants on Mg

11 $${{\rm{M}}}_{2}{{\rm{O}}}_{3}+2{{\rm{Mg}}}_{{\rm{Mg}}}^{{\rm{X}}}\to 2{{\rm{M}}}_{{\rm{Mg}}}^{\bullet }+{{\rm{O}}}_{{\rm{i}}}^{{\prime\prime} }+2{\rm{MgO}}$$

M^3+^ dopants on Ti

12 $${{\rm{M}}}_{2}{{\rm{O}}}_{3}+2{{\rm{Ti}}}_{{\rm{Ti}}}^{{\rm{X}}}+{{\rm{O}}}_{O\,}^{{\rm{X}}}\to 2{{\rm{M}}}_{{\rm{Ti}}}^{^{\prime} }+{{\rm{V}}}_{{\rm{O}}}^{\bullet \bullet }+\,2{{\rm{TiO}}}_{2}$$

13 $${{\rm{M}}}_{2}{{\rm{O}}}_{3}+2{{\rm{Ti}}}_{{\rm{Ti}}}^{{\rm{X}}}+{\rm{MgO}}\to 2{{\rm{M}}}_{{\rm{Ti}}}^{^{\prime} }+{{\rm{Mg}}}_{{\rm{i}}}^{\bullet \bullet }+\,2{{\rm{TiO}}}_{2}$$

**Figure 8:**
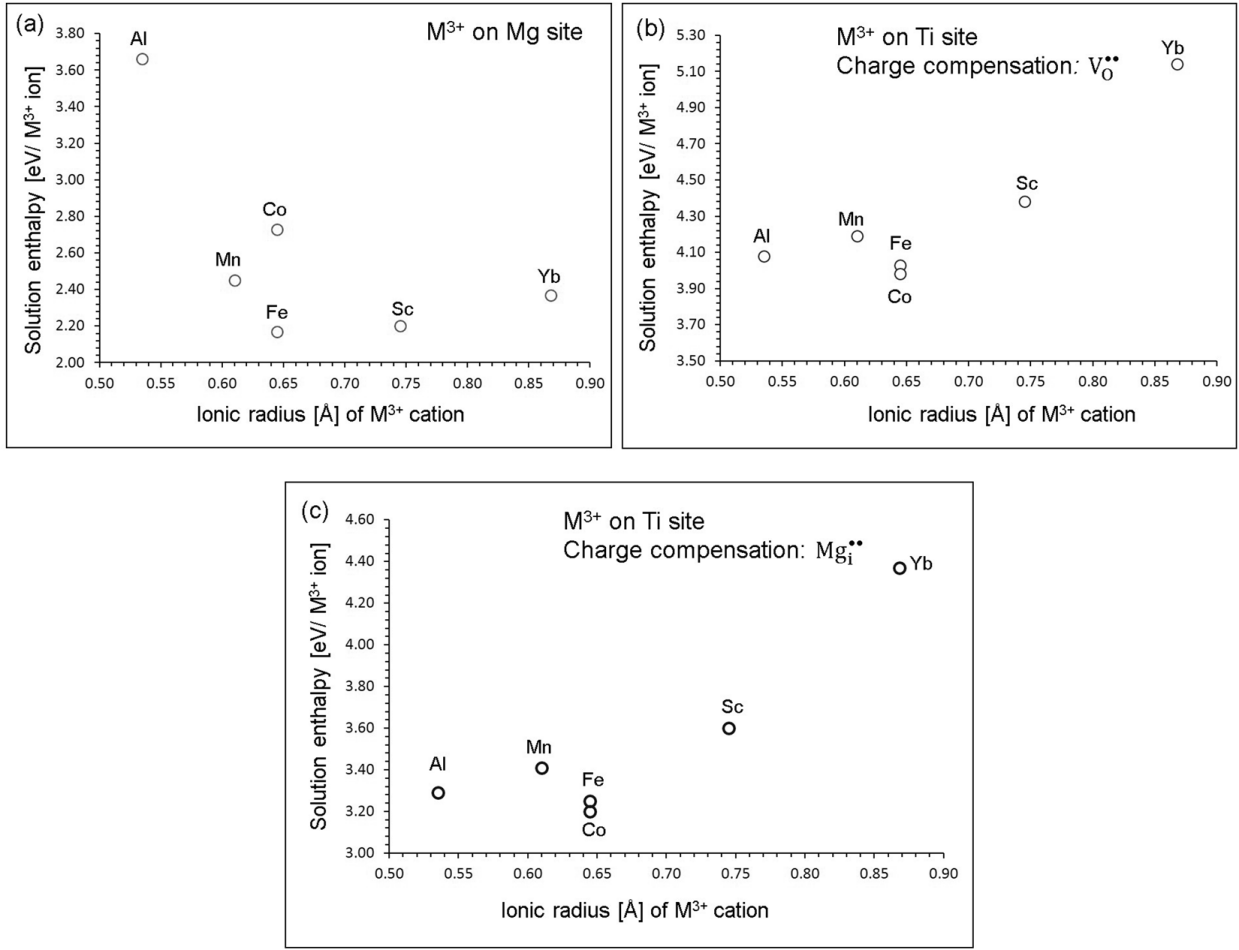
Enthalpy of solution of M_2_O_3_ (M* = *Al, Mn, Co, Mn, Sc and Yb) with respect to the M^3+^ ionic radius in MgTiO_3_.

Doping of M^3+^ ions on Mg site can introduce oxygen interstitials in MgTiO_3_. The most favourable dopant for this process is Fe^3+^ (refer to Fig. 8a). The solution energy for Sc_2_O_3_ is higher only by 0.05 eV/defect compared to that of Fe_2_O_3_. There is an increase in solution energy for dopants Yb^3+^, Mn^3+^ and Co^3+^. Doping of Al^3+^ is predicted to be unlikely. Doping of M^3+^ ions on Ti site can result two possible charge compensations either oxygen vacancies (refer to Equation 12) or magnesium interstitials (refer to Equation 13) in the lattice. In the first charge compensation, the formation of oxygen vacancies is favoured by Co incorporation. Solution energies for dopants Fe, Al and Mn are higher only by at most 0.20 eV than that calculated for Co (refer to Fig. 8b). Yb exhibits an unfavourable solution energy of 5.14 eV/defect. In the second charge compensation, additional Mg ions are introduced in the lattice. This can be an efficient strategy to introduce Mg^2+^ ions to enhance the capacity in the as-prepared MgTiO_3_ material for the applicability of this materials as a viable electrode material in Mg ion batteries. Figure 8c reports the solution energies of M^3+^ dopants on Ti site. The most favourable dopant solution energy (3.20 eV/defect) is calculated for Co^3+^, suggesting that a possible synthesis-doping strategy to introduce additional magnesium into MgTiO_3_ can be achieved by doping Co on Ti sites at elevated temperatures, although the exact amount of Mg incorporation cannot be determined. The possible composition of Mg-doped MgTiO_3_ would be Mg_1+y_Ti_1−y_Co_y_O_3_ (y = 0.0–1.0). Solution energies for Fe_2_O_3_ and Al_2_O_3_ are 3.25 eV/defect and 3.29 eV/defect suggesting that Fe and Al are also promising dopants. High solution energy for Yb_2_O_3_ suggests that Yb^3+^ is an unfavourable dopant to increase Mg^2+^ ions in MgTiO_3_.

Finally, we considered M^4+^ dopants on Mg and Ti sites. The following reaction were constructed using appropriate charge compensating defects:

M^4+^ dopants on Mg site

14 $${{\rm{MO}}}_{2}+{{\rm{Mg}}}_{{\rm{Mg}}}^{{\rm{X}}}\to {{\rm{M}}}_{{\rm{Mg}}}^{\bullet \bullet }+{{\rm{O}}}_{{\rm{i}}}^{{\prime\prime} }+{\rm{MgO}}$$

M^4+^ dopants on Ti site

15 $${{\rm{MO}}}_{2}+{{\rm{Ti}}}_{{\rm{Ti}}}^{{\rm{X}}}\to {{\rm{M}}}_{{\rm{Ti}}}^{{\rm{x}}}+{{\rm{TiO}}}_{2}$$

**Figure 9:**
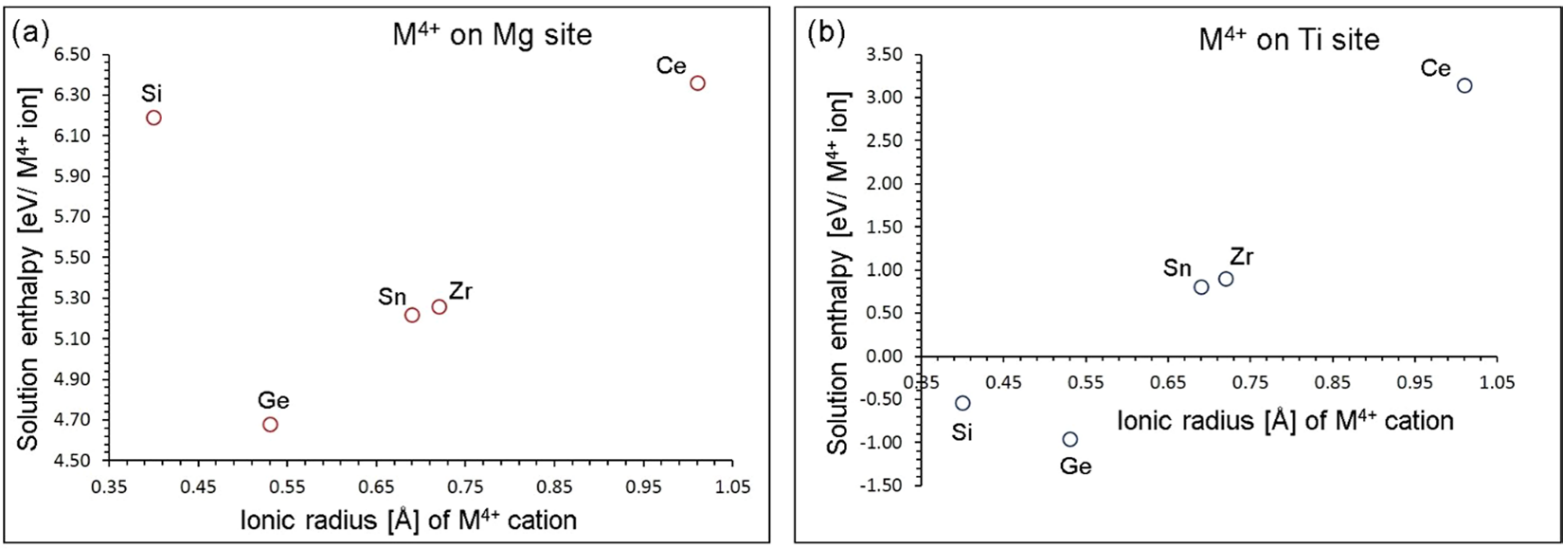
Enthalpy of solution of MO_2_ (M = Si, Ge, Sn, Zr and Ce) with respect to the M^4+^ ionic radius in MgTiO_3_.

The formation of oxygen interstitials via doping of Ge^4+^ on Mg site is the lowest energy process. The higher energies for other dopants suggest that they have relatively lower solubility at the Mg Site. Lower solution energies for isovalent dopants (M^4+^ on Ti site) are observed as no charge compensation is required (refer to Fig. 9). The ionic radius of Ti^4+^ is 0.605 Å. Ionic radii of Si^4+^ and Ge^4+^ are 0.40 Å and 0.53 Å respectively meaning that they can occupy the vacancy position of Ti without gaining energy. This reflects in the exoergic (negative) solution enthalpy. Other dopants have larger ionic radii exhibiting endoergic solution enthalpy. Highest negative solution enthalpy is due to the ionic radius of Ge^4+^ (0.53 Å) closer to the ionic radius of Ti^4+^ (0.605 Å). Both Si and Ge dopants are worth investigating experimentally.

## Summary

In this present study, atomistic simulation technique was employed to understand the defect chemistry and the dopant properties of MgTiO_3_. We predict that the lowest energy disorder is the Mg-Ti anti-site defect. Mg Frenkel is the second lowest energy disorder, although the relative magnitude is relatively high, suggesting that concentration of these defects will not be significant. Considering the vacancy mechanism of ion diffusion, Mg ions migrate via a long range path either along *ab* plane or c axis plane with the overall activation energy of 0.88 eV. Long range O ion conduction will not be significant in this material as overall activation energies are greater than 3 eV. Both divalent and trivalent dopants preferentially occupy the Mg site. Low solution energies calculated for M^2+^ (Co, Mn and Zn) on the Mg site agree with the experimental report. Interestingly, Ge^4+^ and Si^4+^ exhibit negative solution energies on the Ti site suggesting that they are promising dopants for incorporation. Experimental study on this interesting result needs to be explored.

## Methods

The calculations are based on the classical Born model description as implemented in the GULP code^29^. Interactions between ions were modelled using long-range interactions and short-range repulsive interactions representing electron-electron repulsion and van der Waals interactions. Buckingham potentials (Table S1) were used to model short range interactions. Atom positions and simulation boxes were relaxed using the Broyden-Fletcher-Goldfarb-Shanno (BFGS) algorithm^30^. Lattice relaxations around point defects and the migrating ions were modelled using the Mott-Littleton method^31,32^. The Mott-Littleton method partitions the crystal lattice into two spherical regions (region I and region II). Region I contains ions surrounding the defect and those ions were relaxed explicitly. Rest of the crystal (region II) was treated by using approximate quasi-continuum methods. In this region, forces on the defects were relatively weak. In all defect calculations in this study, there were 732 atoms in region I and 5153 atoms in the region II. Activation energy of migration is considered as the local maximum energy along the diffusion path. The defect enthalpies will be overestimated as the present model assumes a full charge ionic model with the calculations corresponding to the dilute limit.

## Supplementary information

Defects, dopants and Mg diffusion in MgTiO3
