# Supplementary material for: Defects, dopants and Mg diffusion in MgTiO3
Source: Sci Rep. 2019 Mar 13;9:4394. doi: 10.1038/s41598-019-40878-y (PMC6416248; doi:10.1038/s41598-019-40878-y)
Supplement: Supplementary file 1 — Defects, dopants and Mg diffusion in MgTiO3 [file 41598_2019_40878_MOESM1_ESM.docx]

**Supporting Information**

**Defects, dopants and Mg diffusion in MgTiO_3_**

Navaratnarajah Kuganathan^1,a)^, Poobalasuntharam Iyngaran^2^, Ruslan Vovk^3^, and Alexander Chroneos^1,4,b)^

^1^Department of Materials, Imperial College London, London, SW7 2AZ, United Kingdom

^2^Department of Chemistry, University of Jaffna, Sir. Pon Ramanathan Road, Thirunelvely, Jaffna, Srilanka

^3^Physics Department, V. Karazin Kharkiv National University, Svobody Sq. 4, 61077 Kharkiv, Ukraine

^4^Faculty of Engineering, Environment and Computing, Coventry University, Priory Street, Coventry CV1 5FB, United Kingdom

Corresponding authors, e-mails: a) n.kuganathan@imperial.ac.uk

b) [alexander.chroneos@imperial.ac.uk](mailto:alexander.chroneos@imperial.ac.uk)

**Table S1**. Interatomic potential parameters used in the atomistic simulations of MgTiO_3_.

Two-body [Φ*_ij_* (*r_ij_*) = *A_ij_* exp (− *r_ij_* /*ρ_ij_*) − *C_ij_ / r_ij_*^6^]

| Interaction | *A* / eV | *ρ* / Å | *C* / eV·Å^6^ | Y / e | K / eV·Å^–2^ | Lattice energy (eV) |
| --- | --- | --- | --- | --- | --- | --- |
| Mg^2+^–O^2−^(1) | 946.627 | 0.31813 | 0.0000 | 2.000 | 999999 | ‒40.23 |
| Ti^4+^–O^2−^(2) | 5111.70 | 0.26250 | 0.0000 | ‒0.100 | 314.0 | ‒124.58 |
| O^2−^–O^2−^(3) | 22764.30 | 0.1490 | 27.879 | -2.86902 | 74.92 | --- |
| Ca^2+^ - O^2−^(4) | 1090.40 | 0.3372 | 0.0000 | 0.7400 | 34.00 | ‒36.77 |
| Sr^2+^ - O^2−^(4) | 1400.00 | 0.3500 | 0.0000 | 0.6700 | 21.53 | ‒33.78 |
| Ba^2+^ - O^2−^(4) | 931.79 | 0.3949 | 0.0000 | 0.5400 | 14.78 | ‒31.33 |
| Mn^2+^ - O^2−^(4) | 715.80 | 0.3464 | 0.0000 | 3.000 | 81.20 | ‒38.38 |
| Fe^2+^ - O^2−^(4) | 694.10 | 0.3399 | 0.0000 | 2.000 | 10.92 | ‒39.51 |
| Co^2+^ - O^2−^(4) | 696.30 | 0.3362 | 0.0000 | 2.000 | 10.74 | ‒40.01 |
| Ni^2+^ - O^2−^(4) | 683.50 | 0.3332 | 0.0000 | 2.000 | 8.77 | ‒40.60 |
| Cu^2+^ - O^2−^(4) | 3799.30 | 0.2427 | 0.0000 | 2.000 | 99999 | ‒44.70 |
| Zn^2+^ - O^2−^(4) | 499.60 | 0.3595 | 0.0000 | 2.050 | 10.28 | ‒39.35 |
| Al^3+^ - O^2−^(4) | 1725.20 | 0.28971 | 0.0000 | 3.000 | 99999 | ‒161.88 |
| Fe^3+^ - O^2−^(4) | 1156.36 | 0.3299 | 0.0000 | 4.970 | 304.70 | ‒148.94 |
| Co^3+^ - O^2−^(4) | 1329.82 | 0.3087 | 0.0000 | 3.000 | 196.30 | ‒157.31 |
| Mn^3+^ - O^2−^(4) | 1267.50 | 0.3214 | 0.0000 | 4.970 | 304.70 | ‒151.46 |
| Sc^3+^ - O^2−^(4) | 1299.40 | 0.3312 | 0.0000 | 3.000 | 99999 | ‒145.27 |
| Yb^3+^ - O^2−^(4) | 1309.60 | 0.3462 | 0.0000 | 3.000 | 99999 | ‒137.45 |
| Sn^4+^ - O^2−^(5) | 1414.32 | 0.3479 | 13.660 | 4.000 | 99999 | ‒113.35 |
| Zr^4+^ - O^2−^(4) | 985.869 | 0.3760 | 0.0000 | 1.350 | 169.617 | ‒109.91 |
| Ce^4+^ - O^2−^(4) | 1986.83 | 0.3511 | 20.40 | 7.700 | 291.75 | ‒105.31 |
| Si^4+^ - O^2−^(6) | 1283.91 | 0.32052 | 10.66 | 4.000 | 99999 | ‒128.73 |
| Ge^4+^ - O^2−^(7) | 1497.3996 | 0.325646 | 16.00 | 4.000 | 99999 | ‒121.58 |

**Table S2.** Energetics of intrinsic defect process in MgTiO_3_

| Defect process/equation | Reaction energy/eV | Reaction energy per defect/eV |
| --- | --- | --- |
| Mg Frenkel /1 | 9.26 | 4.63 |
| Ti Frenkel /2 | 21.40 | 10.70 |
| O Frenkel /3 | 9.36 | 4.68 |
| Schottky /4 | 32.65 | 6.53 |
| MgO Schottky/5 | 10.60 | 5.30 |
| TiO_2_ Schottky/6 | 22.20 | 7.40 |
| Mg/Ti antisite (isolated) /7 | 4.54 | 2.27 |
| Mg/Ti antisite (cluster) /8 | 0.84 | 0.42 |

**Table S3**. Calculation formulas for intrinsic and extrinsic defect processes.

| **Defect process** | **Equation number** | **Calculation formula** |
| --- | --- | --- |
| $Mg Frenkel$ | 1 | $E(V_{\mathrm{Mg}}^{''})+ {E(Mg}_{i}^{\bullet⦁})$ |
| $O Frenkel$ | 2 | $E(V_{O}^{\bullet\bullet})+ E(O_{i}^{''})$ |
| $Ti Frenkel$ | 3 | $E(V_{\mathrm{Ti}}^{''''})+ E(\mathrm{Ti}_{i}^{\bullet\bullet\bullet\bullet})$ |
| $\mathrm{Schottky}$ | 4 | $E\left( V_{\mathrm{Mg}}^{''} \right)+ E\left( V_{\mathrm{Ti}}^{''''} \right)+3E\left( V_{O}^{\bullet\bullet} \right)+E_{\mathrm{lat}}(MgTiO_{3})$ |
| $MgO Schottky$ | 5 | $E{(V}_{\mathrm{Mg}}^{''})+E(V_{O}^{\bullet\bullet})+ E_{\mathrm{lat}}(MgO)$ |
| $\mathrm{Ti}O_{2}\mathrm{Schottky}$ | 6 | $E(V_{\mathrm{Ti}}^{''''})+2 {E(V}_{O}^{\bullet\bullet})+ E_{\mathrm{lat}}(TiO_{2})$ |
| $Mg/Ti antisite (isolated)$ | 7 | $E\left( \mathrm{Mg}_{\mathrm{Ti}}^{''} \right)+E(\mathrm{Ti}_{\mathrm{Mg}}^{\bullet\bullet})$ |
| $Mg/Ti antisite (cluster)$ | 8 | $E \{(\mathrm{Mg}_{\mathrm{Ti}}^{''}:\mathrm{Ti}_{\mathrm{Mg}}^{\bullet\bullet})$^X^} |
| M^2+^ on Mg site | 9 | $E\left( \mathrm{MgO} \right)-E(\mathrm{MO})$ |

**Table S4**. Solution enthalpy for dopant substation in MgTiO_3_

| Dopant | Solution enthalpy (eV/dopant) | |
| --- | --- | --- |
|  | Mg site | Ti site |
| Ca^2+^ | 0.29 | 9.38 |
| Sr^2+^ | 1.64 | 10.91 |
| Ba^2+^ | 3.64 | 12.62 |
| Mn^2+^ | 0.11 | 8.88 |
| Fe^2+^ | 0.14 | 8.75 |
| Co^2+^ | 0.20 | 8.72 |
| Ni^2+^ | 0.45 | 8.71 |
| Cu^2+^ | 1.00 | 8.97 |
| Zn^2+^ | 0.24 | 8.71 |
| Al^3+^ | 3.66 | 4.08 |
| Fe^3+^ | 2.17 | 4.03 |
| Co^3+^ | 2.73 | 3.98 |
| Mn^3+^ | 2.45 | 4.19 |
| Sc^3+^ | 2.20 | 4.38 |
| Yb^3+^ | 2.37 | 5.14 |
| Sn^4+^ | 5.22 | 0.81 |
| Zr^4+^ | 5.26 | 0.90 |
| Ce^4+^ | 6.36 | 3.14 |
| Si^4+^ | 6.19 | ‒0.54 |
| Ge^4+^ | 4.68 | ‒0.96 |

**References**

1 Heath, J., Chen, H. & Islam, M. S. MgFeSiO_4_ as a potential cathode material for magnesium batteries: ion diffusion rates and voltage trends. *J. Mater. Chem. A* **5**, 13161-13167 (2017).

2 Olson, C. L., Nelson, J. & Islam, M. S. Defect Chemistry, Surface Structures, and Lithium Insertion in Anatase TiO_2_. *J. Phys. Chem. B* **110**, 9995-10001(2006).

3 Gale, J. D. & Henson, N. J. Derivation of interatomic potentials for microporous aluminophosphates from the structure and properties of berlinite. *J. Chem. Soc. Faraday Trans.* **90**, 3175-3179 (1994).

4 Tealdi, C., Saiful Islam, M., Malavasi, L. & Flor, G. Defect and dopant properties of MgTa_2_O_6_. *J. Solid State Chem.* **177**, 4359-4367 (2004).

5 Minervini, L., Grimes, R. W. & Sickafus, K. E. Disorder in Pyrochlore Oxides. *J. Am. Ceram. Soc.* **83**, 1873-1878 (2000).

6 Kuganathan, N. & Islam, M. S. Li_2_MnSiO_4_ Lithium Battery Material: Atomic-Scale Study of Defects, Lithium Mobility, and Trivalent Dopants. *Chem. Mater.* **21**, 5196-5202 (2009).

7 Kendrick, E., Islam, M. S. & Slater, P. R. Atomic-scale mechanistic features of oxide ion conduction in apatite-type germanates. *ChemComm*, 715-717 (2008).
